# Effect of organ failure on outcomes in neutropenic sepsis

**DOI:** 10.1186/cc9708

**Published:** 2011-03-11

**Authors:** D Bareisiene, R Kapoor

**Affiliations:** 1 East Kent Hospitals University NHS Foundation Trust, Canterbury, UK

## Introduction

The objective was to assess correlation between organ failure and outcomes in patients admitted with neutropenic sepsis to an adult ICU in a district general hospital.

## Methods

Retrospective data were collected for admissions with neutropenic sepsis to the ICU over a 3-year period. The Ward Watcher electronic system was used to collect data on the level of organ support on the ICU. Outcomes assessed were 30-day and 1-year mortality.

## Results

**Figure 1:**
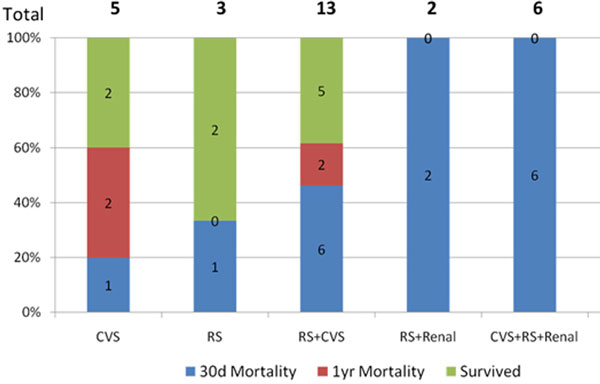
**Outcomes depending on support level**.

Twenty-nine neutropenic patients were admitted during the study period; 93% had haematological malignancy while 7% showed no evidence of malignancy. The mean neutrophil count was 0.2 × 10^9^/l and 52% had zero count during their ICU stay. A total of 41.3% had positive blood cultures. Mortality with negative blood cultures was 73%. Overall 30-day mortality was 58.6% and 1-year mortality was 79.3%. Ventilator support was needed in 83% with a mortality of 88%. Inotropes were required in 48.2% and there was a 71% 30-day mortality. Renal support was commenced in 27.5% with 100% mortality. The 30-day mortality was 100% in patients requiring invasive ventilation and renal support. Mortality was also 100% in those requiring three-organ support (Figure 1).

## Conclusions

Our data suggest a significant mortality in mechanically ventilated patients with neutropenic sepsis. This rises to 100% if two or more organs are supported, especially if one of them is the kidney. Early recognition and intervention to prevent progression to multiorgan failure is paramount to improve outcomes.
